# Retraction Note: Double-negative metamaterial square enclosed Q.S.S.R For microwave sensing application in S-band with high sensitivity and Q-factor

**DOI:** 10.1038/s41598-026-49765-9

**Published:** 2026-04-28

**Authors:** Muhammad Amir Khalil, Wong Hin Yong, Mohammad Tariqul Islam, Ahasanul Hoque, Md. Shabiul Islam, Cham Chin leei, Mohamed S. Soliman

**Affiliations:** 1 Faculty of Engineering (F.O.E.), Multimedia University (MMU), 63100, Cyberjaya, Selangor Malaysia; 2 Department of Electrical, Electronic and Systems Engineering, Faculty of Engineering and Built Environment, University Kebangsaan Malaysia, 43600 Bangi, Malaysia; 3 Institute of Climate Change, University Kebangsaan Malaysia, 43600 Bangi, Malaysia; 4 Department of Electrical Engineering, College of Engineering, Taif University, P.O. Box 11099, 21944 Taif, Saudi Arabia

Retraction of: *Scientific Reports* 10.1038/s41598-023-34514-z, published online 05 May 2023

The Editors have retracted this Article.

Following publication, concerns were raised about the analyses and some of the claims of this Article. A post-publication peer review confirmed that the Q-factor reported in the paper is incorrect, which invalidates the conclusions. The Editors have therefore lost confidence in the claims of this Article.

All Authors disagree with this retraction.

